# Obesity resistant mechanisms in the Lean polygenic mouse model as indicated by liver transcriptome and expression of selected genes in skeletal muscle

**DOI:** 10.1186/1471-2164-12-96

**Published:** 2011-02-03

**Authors:** Matjaž Simončič, Tadeja Režen, Peter Juvan, Damjana Rozman, Gregor Fazarinc, Catherine Fievet, Bart Staels, Simon Horvat

**Affiliations:** 1 University of Ljubljana, Biotechnical Faculty, Department of Animal Science, Groblje 3, 1230 Domžale, Slovenia; 2 University of Ljubljana, Institute of Biochemistry, Medical Faculty, Vrazov trg 2, 1000 Ljubljana, Slovenia; 3 Institute of Anatomy, Histology and Embryology, Veterinary faculty, University of Ljubljana, Gerbičeva 60, 1000 Ljubljana, Slovenia; 4 University Lille Nord de France, F-59000, Lille, France; 5 Inserm, U1011, F-59000, Lille, France; 6 UDSL, F-59000, Lille, France; 7 Institut Pasteur de Lille, F-59019, Lille, France; 8 National Institute of Chemistry, Hajdrihova 19, SI-1001 Ljubljana, Slovenia

## Abstract

**Background:**

Divergently selected Lean and Fat mouse lines represent unique models for a polygenic form of resistance and susceptibility to obesity development. Previous research on these lines focused mainly on obesity-susceptible factors in the Fat line. This study aimed to examine the molecular basis of obesity-resistant mechanisms in the Lean line by analyzing various fat depots and organs, the liver transcriptome of selected metabolic pathways, plasma and lipid homeostasis and expression of selected skeletal muscle genes.

**Results:**

Expression profiling using our custom Steroltalk v2 microarray demonstrated that Lean mice exhibit a higher hepatic expression of cholesterol biosynthesis genes compared to the Fat line, although this was not reflected in elevation of total plasma or liver cholesterol. However, FPLC analysis showed that protective HDL cholesterol was elevated in Lean mice. A significant difference between the strains was also found in bile acid metabolism. Lean mice had a higher expression of *Cyp8b1*, a regulatory enzyme of bile acid synthesis, and the *Abcb11 *bile acid transporter gene responsible for export of acids to the bile. Additionally, a higher content of blood circulating bile acids was observed in Lean mice. Elevated HDL and upregulation of some bile acids synthesis and transport genes suggests enhanced reverse cholesterol transport in the Lean line - the flux of cholesterol out of the body is higher which is compensated by upregulation of endogenous cholesterol biosynthesis. Increased skeletal muscle *Il6 *and *Dio2 *mRNA levels as well as increased activity of muscle succinic acid dehydrogenase (SDH) in the Lean mice demonstrates for the first time that changes in muscle energy metabolism play important role in the Lean line phenotype determination and corroborate our previous findings of increased physical activity and thermogenesis in this line. Finally, differential expression of *Abcb11 *and *Dio2 *identifies novel strong positional candidate genes as they map within the quantitative trait loci (QTL) regions detected previously in crosses between the Lean and Fat mice.

**Conclusion:**

We identified novel candidate molecular targets and metabolic changes which can at least in part explain resistance to obesity development in the Lean line. The major difference between the Lean and Fat mice was in increased liver cholesterol biosynthesis gene mRNA expression, bile acid metabolism and changes in selected muscle genes' expression in the Lean line. The liver *Abcb11 *and muscle *Dio2 *were identified as novel positional candidate genes to explain part of the phenotypic difference between the Lean and Fat lines.

## Background

The 20th century is marked by an explosive increase in obesity and metabolic syndrome commencing in populations of the Western world in the 1960's and is becoming a significant burden also in developing nations [1]. Metabolic syndrome encompasses a cluster of deleterious metabolic events characterized by obesity, hyperglycemia, insulin resistance, hypercholesterolemia, hypertriglyceridemia and hypertension. Environmental determinants influencing the development of the metabolic syndrome include low-cost highly palatable foods and the increasing mechanization of transport and manual tasks following the industrial revolution. While the intake of calories gradually declined over the past decades, physical inactivity and its interaction with genetic predisposition to obesity seem to be an increasingly prevalent determinant of metabolic syndrome development [2,3].

Previous research in the field of metabolic syndrome and obesity primarily studied monogenic knockout rodent models [4]. Although murine models with single-gene mutations are useful to study the effects of a few genes with major effect on human obesity, monogenic defects account for only a minority of patients with morbid obesity. In contrast, epidemic proportions of the metabolic syndrome and obesity are ascribed to the polygenic effects, where multiple genes interact with many environmental factors over time [5,6]. Thus, single gene animal models can not explain a large portion of genetic variation that exists in the polygenic form making polygenic obesity mouse models [7,8], such as the lines used herein, a more suitable genetic resource.

Accordingly, we employed Lean and Fat mouse lines developed from the same base population by long-term (over 60 generations) divergent selection for low or high body fat % [9]. These Fat and Lean mouse lines may represent a unique model of the prevalent polygenic form of human obesity and leanness. Because previous research focused mainly on investigating obesity-susceptibility in the Fat line (e.g., [10-12]), we here aimed to elucidate the molecular basis of obesity-resistance in the Lean line.

We performed a thorough phenotypic characterization of the Lean and Fat lines. In addition, a gene-expression analysis was conducted on the liver tissue using the Steroltalk v2 microarray that was previously used to study hepatic lipid homeostasis [13-15]. This custom microarray is designed for detailed studies of genes from the cholesterol homeostasis and drug metabolism networks, including bile acid metabolism, and several genes from the fatty acid and glucose pathways as well as related transporters for the aforementioned processes.

The results of our study combined with previous findings help to identify, at least in part, a molecular basis for the contrasting phenotype between the Lean and Fat mice and substantiate a hypothesis that differences in metabolic interactions between skeletal muscle and liver tissues play an important part in the phenotype divergence between these lines. The Lean mouse line exhibits characteristics that uncover some of the factors involved in genetic predisposition for obesity-resistance that may also be relevant for obesity and metabolic syndrome basic and applied research in humans.

## Methods

### Animals and maintenance

Inbred lean (Lean) and fat (Fat) mouse lines used here were developed by long-term (over 60 generations) divergent selection for low or high body fat % [9,16]. Our previous genetic studies demonstrated that these lines differ in large part due to cumulative action of multiple quantitative trait loci (QTLs) of small to medium sized effects, confirming that these lines may present a valuable model of the polygenic form of leanness/obesity [17-19].

Animals were weaned at 3 weeks of age and one pair of males (usually brothers) was housed per cage. Cages were randomly distributed on the rack in order to minimize localized environmental effects and mice were maintained at 21.5°C (± 0.5) with 12 h light/dark cycle. Mice were fed maintenance chow diet (Altromin 1324, Lage, Germany), containing: crude oil, 4%; crude protein, 19%; crude fibre, 6%; ash, 7%; moisture, 13.5%; metabolizable energy, 11.9 MJ/kg where 24 %, 11 % and 65 % of calories from protein, fat and carbohydrates, respectively. Body weights were recorded at 3, 6, 10 and 14 weeks of age. All procedures on animals were performed according to local ethical and regulatory guidelines, which are all in compliance with the EU regulations regarding research on experimental animals.

### Phenotype measurements and analyses

At 14 weeks of age (± 3 days) a total of 43 Lean and 42 Fat males were sacrificed after four hour fast by decapitation. Samples of blood, liver, entire left epididymal, left abdominal (perirenal and retroperitoneal), left femoral fat, mesenterial fat, heart and striated muscle from *regio cruris *(*m.gastrocnemius*) and thigh muscle (*m. quadriceps*) were weighed, promptly frozen in liquid nitrogen and stored at -80°C.

Blood was collected in Li-heparin tubes (Sarstedt, Germany) and centrifuged within 3 h of collection at 3000 rpm/min, for 10 min at +4°C. Plasma samples of 10 Lean and 10 Fat mice were individually analyzed for total plasma cholesterol, HDL-cholesterol, plasma triglyceride and glucose concentrations. Plasma total cholesterol and triglyceride concentrations were determined by enzymatic assays adapted to microtiter plates using commercially available reagents (BioMerieux, Lyon, France). Plasma HDL-cholesterol levels were measured after precipitation of apolipoprotein (Apo) B-containing lipoproteins with phosphotungstic acid/Mg (Roche Diagnostics GmbH, Mannheim, Germany). Non-HDL cholesterol was obtained by subtraction of HDL-cholesterol values from total plasma cholesterol.

Plasma lipoproteins were separated by gel filtration chromatography using a Superose 6 HR 10/30 column (Pharmacia, Sweden). The gel was allowed to equilibrate with 10 mM phosphate-buffered saline (PBS) containing 0.01% (wt/vol) EDTA and 0.01% (wt/vol) sodium azide. Plasma was eluted with the buffer at room temperature at a flow rate of 0.2 ml per minute. The effluents were collected in 0.22 ml fractions. Cholesterol and triglyceride concentrations were determined in the eluted fractions as described above.

Cholesterol distribution among lipoproteins was obtained by separation of the major lipoprotein classes (VLDL, IDL+LDL, and HDL) by fast protein liquid chromatography (FPLC) carried out on 200 μl of plasma sample for Fat and Lean line, according to procedure previously detailed [12]. Cholesterol concentrations were determined in the eluted fractions.

Analysis of hepatic lipids in individual samples of 10 Lean and 10 Fat mice was carried out using frozen liver tissue (50 mg) which was homogenized in SET buffer (1 mL ; sucrose 250 mM, EDTA 2 mM and Tris 10 mM), followed by two freeze-thaw cycles and three passages through a 27-gauge syringe needle and a final freeze-thaw cycle to ensure complete cell lysis. Protein content was determined with the BCA method and triglyceride and cholesterol was measured as described above.

Differences between the lines in all measured phenotypic parameters were tested by an unpaired Student *t*-test on untransformed data as the data exhibited normal distribution.

### SDH activity of striated muscles

Oxidative capacity of the muscle fibres (mid-portion of *m. gastrocnemius*) was determined by the mitochondrial succinate dehydrogenase (SDH) assay as described previously [20]. Briefly, 10 μm thick cryostat sections were incubated for 45 minutes at 37°C in a moisture chamber with the medium composed of equal volumes of 0.2 M phosphate buffer saline (pH 7.6), 0.2 M disodium succinate and aqueous solution of nitro-BT (1 mg/ml). After incubation the slides were washed in tap water, dehydrated and mounted with Synthetic Mountant (Shandon, USA). SDH has an important role in metabolism of tricarbonic acids by the Krebs cycles. In the histochemical reaction the enzyme takes over the hydrogen from disodium succinate and transfers it to the tetrasolium salt (NBT), which turns visible in the form of blue formazan granules.

To analyze the SDH activity in muscle fibres a Nikon Microphot FXA microscope (Nikon Instruments Europe BV, Badhoevendorp, The Netherlands) and the Lucia-G analyzing software (Laboratory Imaging, Prague, Czech Republic) were used. In total 12 of each Lean and Fat line mice were analyzed. 150 muscle fibres of each mouse were measured by densitometry. Higher % densitometry indicated higher transparency resulting from lower SDH-enzyme activity.

### RNA isolation

10 liver samples were individually homogenized for 10 s using an Ultra-Turrax T8 homogenizer (IKA Labortechnik, Germany) and RNA extracted in »batches« (a pair of Fat and Lean line samples simultaneously) using TRIzol reagent (TRIzol^® ^reagent, Invitrogen, Life technologies). RNA quality was evaluated by ND-1000 spectrophotometer (Nanodrop Technologies, Wilmington, USA) and Agilent 2100 bioanalyzer. According to the given RINs (RNA integrity number) a pairwise comparison between Fat and Lean line RNA samples was made in order to couple samples with least quality divergence. RIN values of all samples ranged from 7.5-8.5 suggesting low variability and high overall quality. Maximal tolerated RNA quality divergence for a pair of Lean and Fat-line samples co-hybridized on the same microarray was ± 0.5 RIN.

### Microarray experiment

The Steroltalk v2 cDNA microarray used here was a next generation of the previously described Sterolgene v0 array [14] with additional cDNA probes detecting a total of 278 mouse genes from selected metabolic pathways relevant to obesity research. The design and preparation of these additional probes was the same as described [14]. The Steroltalk v2 cDNA microarray was focused on targeted functional groups such as enzymes from cholesterol biosynthesis (18/21 probes; coverage 85.7%), nuclear receptor families (45/49 probes; coverage 93.75%; http://www.nursa.org/) and members of the cytochrome P450 (38/103 probes, coverage 36.9%) [14]. Additionally, key enzymes of glucose and fatty acid metabolism; transporters of xenobiotics, cholesterol and bile acids; and proteins involved in cholesterol plasma transport, circadian rhythm, and selected cell signalling pathways involved in the regulation of cholesterol homeostasis were included. A full list of genes contained on the custom Steroltalk v2 array along with associated functional groups is provided in Additional file 1.

Individual total RNA samples from 10 Lean and 10 Fat-line animals were labelled using Cy3 and Cy5 dye. To 20 μg of Lean and Fat line total RNA sample, spike of control RNAs was added: 250 pg of Firefly Luciferase mRNA (Promega, Madison, WI, USA) and 0.5 μL of either test or reference spike mix from Lucidea Universal Scorecard kit (Amersham Biosciences, GE Healthcare UK limited, Little Chalfont, UK). mRNA was reverse transcribed to amino-allyl cDNA using 2.5 μg of Oligo dT (Invitrogen, Carlsbad, CA, USA), 400 U of SuperScript™ III Reverse Transcriptase (Invitrogen, Carlsbad, CA, USA) and 1 μL of 10 mM amino-allyl dUTP (Sigma, St Louis, MI, USA) according to the manufacturer's protocol. Reactions were stopped after 2 hours by addition of 10 μL of 0.5 M EDTA and of 1 M NaOH and were further incubated at 65°C for 15 min. 10 μL of 1 M HCl was added and cDNA was purified using MinElute PCR Purification Kit (Qiagen GmBH, Hilden, D) according to the manufacturer's protocol with exception of using a phosphate buffer (5 mM KPO_4 _pH 8.5 in 80% ethanol) for the washing step and MilliQ water for elution. Purified amino-allyl cDNA was dried and resuspended in 4.5 μL of 0.2 M Na_2_CO_3 _(pH 9.0) and 4.5 μL of cyanin-3 or cyanin-5 dye in DMSO (Amersham Biosciences, GE Healthcare UK limited, Little Chalfont, UK). The labelling reaction was incubated at room temperature for two hours, precipitated by addition of 35 μL of 0.1 M Na acetate (pH 5.2) and purified reaction using a MinElute PCR Purification Kit (Qiagen GmBH, Hilden, D) were added. Labelled cDNA was eluted in water and 1 μL was used for evaluation on ND-1000 spectrophotometer (Nanodrop Technologies, Wilmington, Delaware, USA). 10 Lean and Fat line cDNA samples, respectively, were co-hybridized in pairs and dye swaps were performed. Using LifterSlip cover glasses (Erie Scientific Company, Portsmouth, NH, USA) samples were hybridized for 16 h at 65°C using buffers 3×SSC and 0.2% SDS in humidified hybridization chambers (HybChambers, GeneMachines, San Carlos, CA, USA). The slides were washed and scanned as described [14].

Images were analyzed using Array-Pro Analyzer 4.5 (Media Cybernetics, Bethesda, MD, USA). Three arrays did not pass quality control parameters (mainly too high signal to noise ratios) and hence 17 arrays were included in downstream statistical analyses. Data were normalized using OWNormalize, a widget for explorative normalization of focused microarrays, implemented within Orange software [21]. Data were first filtered to exclude spots of low quality and then normalized using LOWESS fit to "spike in" control RNAs (Firefly luciferase and Lucidea Universal Scorecard) according to their average intensity. The raw and normalized gene expression data of 17 arrays together with experimental information are deposited in Gene Expression Omnibus database (http://www.ncbi.nlm.nih.gov/geo/, accession number GSE24967) in compliance with MIAME standards [22]. Differential expression was assessed using Student's t-test at probability of type I error α=0.05. Expression of genes was considered on individual basis and therefore no multiple testing correction has been applied.

### Quantitative reverse transcription polymerase chain reaction (qRT-PCR)

**Figure 1:**
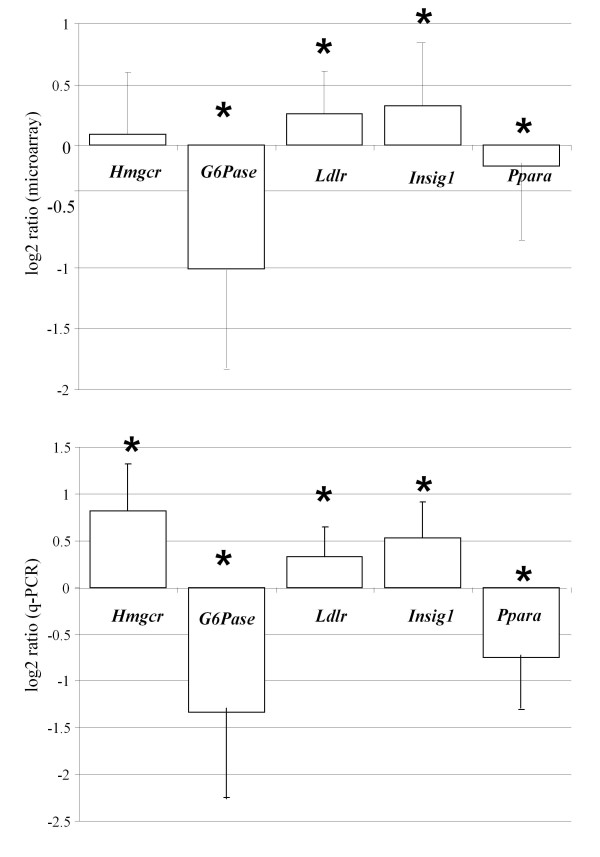
**Comparison of randomly selected genes in the microarray experiment and by qRT-PCR method**. Five genes shown to be differentially expressed by the microarray analysis (upper panel) were randomly selected for validation by qRT-PCR (lower panel). All five genes also showed statistical significant differences (*P < 0.05) between the Lean and Fat line by qRT-PCR as well as the same direction of gene-expression differences supporting the credibility of microarray experiment. Average log_2 _ratios +/- standard errors are displayed.

A random selection of differentially expressed genes from the microarray experiment and some genes not represented on the array (for assaying in muscle tissue) were assayed by the qRT-PCR to validate array results (Table 1 Table 2, and Figure 1). The same liver RNAs were used as originally in the array experiments. In analyses of muscle genes, 8 and 10 RNAs from the Lean and Fat were used, respectively - again, using the same animals as used in the array experiment. Briefly, 1 μg of total RNA was DNAse I (Sigma, St Louis, MI, USA) treated, reverse-transcribed (SuperScript™ III, Invitrogen, USA) using random primers (Promega, USA) and Platinum^® ^SYBR^® ^Green qRT-PCR SuperMix-UDG (Invitrogen, Carlsbad, CA, USA) and analyzed on an ABI PRISM 7900 HT (PE Applied Biosystems, Foster City, CA, USA) according to the manufacturer's protocols. Internal controls were *18 S rRNA *for liver and *Actb *(beta-actin) for muscle. Relative transcript levels were statistically analyzed using the comparative Ct (cycle threshold) and -ΔΔCt values [23]. Single-factor analysis of variance and a probability of type I error α=0.05 was used to determine statistical significance in SPSS 14.0 (SPSS Inc., Chicago, Illinois, USA). Primer sequences are provided in Table 1.

**Table 1:** Primer sequences used in quantitative qRT-PCR analyses

Gene name	Gene symbol	Sense primer (5'-3')	Antisense primer (5'-3')
3-hydroxy-3-methylglutaryl-Coenzyme A reductase	*Hmgcr*^1^	cttgtggaatgccttgtgattg	agccgaagcagcacatgat
Low density lipoprotein receptor	*Ldlr*^1^	aggctgtgggctccatagg	tgcggtccagggtcatct
Insulin induced gene 1	*Insig1 *^2^	tcacagtgactgagcttcagca	tcatcttcatcacacccaggac
Peroxisome proliferator activated receptor α	*Ppara *^3^	cctcttcccaaagctccttca	cgtcggactcggtcttcttg
Deiodinase iodothyronine type II	*Dio2^4^*	Mm00515664_m1	
Interleukin-6	*Il6^4^*	Mm00446190_m1	
Glucose-6 phosphatase	*G6Pc^4^*	Mm00839363_m1	
18 S ribosomal RNA	*18S*^5^	cgccgctagaggtgaaattc	ttggcaaatgctttcgctc
β-actin	*Actb*	ccgtgaaaagatgacccagatc	cacagcctggatggctacgt

^1 ^Reference [41]; ^2 ^Reference [42]; ^3 ^Reference [43] ; ^4 ^Assay-on-demand (Applied Biosystems) ^5 ^Reference [44]

**Table 2:** Differential gene expression in skeletal muscle between the Lean and Fat mice

SKELETAL MUSCLE (gene name)	Gene symbol	log_2 _ratio (qRT-PCR) *	L:F**
Interleukin-6	*Il6*	0.71*	↑
Deiodinase iodothyronine type II	*Dio2*	-0.5*	↓

*P < 0.05

**Up arrow indicates increased mRNA expression in the Lean (L) line and down arrow decreased expression in the Lean line compared to the Fat (F) line

## Results

### Lean mice exhibit a liver transcriptome profile suggesting improved cholesterol homeostasis, bile acid, glucose and lipoprotein metabolism

Analysis of Steroltalk v2 microarray data identified 38 differentially expressed genes (P < 0.05) that were further separated into metabolic pathway groups such as cholesterol biosynthesis pathway, bile and glucose metabolism, drug metabolism and others (Table 3). Gene-expression differences as well as their statistical significances were confirmed by qRT-PCR (Figure 1). Expression of several genes involved in the cholesterol biosynthesis pathway was found to be elevated in Lean mice (Table 3), suggesting that liver cholesterol production might also be elevated compared to the Fat mice. In addition, the *Insig1 *gene, a potent regulator of cholesterol biosynthesis transcription factors and a modulator of HMG-CoA reductase protein ubiquitination, was also expressed at a higher level in Lean mice (Table 3).

**Table 3:** Differential expression between the Lean and Fat mice in liver genes

Gene name	Gene Symbol	GeneBank Code	Log_2 _ratio*	L:F**
**Cholesterol metabolism**				
Mevalonate kinase	*Mvk*	NM_023556	0.11	↑
NAD(P) dependent steroid dehydrogenase-like	*Nsdhl*	BC019945	0.15	↑
Sterol-C5-desaturase (fungal ERG3, delta-5-desaturase) homolog (S. cerevisae)	*Sc5d*	BC024132	0.27	↑
Mevalonate (diphospho) decarboxylase	*Mvd*	NM_138656	0.29	↑
Lanosterol synthase	*Lss*	NM_146006	0.33	↑
Farnesyl diphosphate synthetase	*Fdps*	NM_134469	0.41	↑
Farnesyl diphosphate farnesyl transferase 1	*Fdft1*	NM_010191	0.44	↑
Acyl-CoA synthetase short-chain family member 2	*Acss2*	NM_019811	0.36	↑
***Bile acid and glucose metabolism***				
Glucose-6-phosphatase, catalytic	*G6Pc*	NM_008061	-1.01	↓
Pyruvate dehydrogenase (lipoamide) beta	*Pdhb*	NM_024221	-0.28	↓
Solute carrier organic anion transporter family, member 1a4	*Slco1a4*	NM_030687	-0.71	↓
Solute carrier organic anion transporter family, member 1a1	*Slco1a1*	AY195868	-0.49	↓
Solute carrier organic anion transporter family, member 1b2	*Slco1b2*	NM_020495	-0.36	↓
Solute carrier family 10 (sodium/bile acid cotransporter family), member 1	*Slc10a1*	BC094023	-0.13	↓
ATP-binding cassette, sub-family G (WHITE), member 5	*Abcg5*	NM_031884	-0.15	
ATP-binding cassette, sub-family B (MDR/TAP), member 11	*Abcb11*	NM_021022	0.65	↑
Cytochrome P450, family 8, subfamily b, polypeptide 1	*Cyp8b1*	NM_010012	0.52	↑
**Drug metabolism**				
Cytochrome P450, family 2, subfamily a, polypeptide 5	*Cyp2a5*	BC011233	-0.52	↓
Cytochrome P450, family 2, subfamily b, polypeptide 10	*Cyp2b10*	AK028103	-0.44	↓
Cytochrome P450, family 2, subfamily b, polypeptide 9	*Cyp2b9*	NM_010000	-0.38	↓
Cytochrome P450, family 2, subfamily b, polypeptide 9	*Cyp2b9*	NM_007813	-0.38	↓
Cytochrome P450, family 3, subfamily a, polypeptide 41A	*Cyp3a41a*	NM_017396	-0.37	↓
Cytochrome P450, family 2, subfamily c, polypeptide 69	*Cyp2c69*	NM_010004	0.34	↑
Cytochrome P450, family 1, subfamily a, polypeptide 2	*Cyp1a2*	NM_009993	0.41	↑
**Others**				
Complement component 9	*C9*	BC011137	-1.37	↓
Serum amyloid P-component	*Apcs*	BC061125	0.46	↑
Complement component 4B (Childo blood group)	*C4b*	BC067409	1.55	↑
C-reactive protein, pentraxin-related	*Crp*	NM_007768	0.25	↑
Orosomucoid 1	*Orm1*	BC012725	-0.34	↓
Hemopexin	*Hpxn*	BC019901	0.12	↑
Nuclear receptor subfamily 5, group A, member 2 (LRH-1)	*Nr5a2*	NM_030676	0.16	↑
Acetyl-Coenzyme A acetyltransferase 2	*Acat2*	NM_009338	0.22	↑
Apolipoprotein A-II	*Apoa2*	BC031786	0.23	↑
Lysosomal acid lipase A	*Lip1*	NM_021460	0.23	↑
Low density lipoprotein receptor	*Ldlr*	BC019207	0.27	↑
Insulin induced gene 1	*Insig1*	NM_153526	0.33	↑
Nitric oxide synthase 1, neuronal	*Nos1*	BC066101	0.34	↑
Adiponectin receptor 2	*Adipor2*	NM_197985	0.35	↑

*Data represent log2 ratios of Lean (L) versus Fat (F) line (p < 0.05*)*.

**Up arrow indicates increased mRNA expression of particular gene in the Lean (L) line and down arrow decreased expression in the Lean line.

Transporter genes involved in the uptake of plasma bile acids into hepatocytes such as *Slco1a1, Slco1a4, Slco1b2, Slc10a1*, and the cholesterol efflux transporter *Abcg5 *were down-regulated in Lean mice. However, *Abcb11*, which controls bile acid excretion, was up-regulated. *Cyp8b1*, a gene encoding a bile acid synthesis enzyme, was also expressed at a higher level in Lean mice (Table 3).

Gluconeogenesis genes *G6Pc (*glucose-6-phosphatase, catalytic) and *Pdhb *(pyruvate dehydrogenase beta) were markedly down-regulated in Lean mice supporting previously identified predisposition of Lean line to normoglycemia and resistance to diabetes development (Table 3).

The *Ldlr, Lip1 *and *Adipor2 *receptors were up-regulated in livers of Lean mice suggesting that livers of Lean mice have an increased ability to capture atherogenic LDL-cholesterol as well as to respond to the white adipose tissue derived hormone adiponectin. In contrast, the transcription factor *Ppara*, involved in triglyceride homeostasis, was down-regulated in Lean mice (Table 3).

Many of the cytochromes P450 functioning in drug and steroid hormone metabolism were down-regulated (*Cyp2a5*, *Cyp2b10*, *Cyp2b9 *and *Cyp3a41*) although two (*Cyp2c69 *and *Cyp1a2*) were up-regulated (Table 3). However, it is important to note that the cDNA probes used on the microarrays do not enable efficient differentiation between the aforementioned CYP members, which share a relatively high sequence homology. For example, probes for *Cyp2b10 *and *Cyp2b9 *cross-react with other *Cyp2b *family members, and the same holds true for the *Cyp2c*, *Cyp3a *and *Cyp2a *probes. Therefore, to determine exactly which gene from the CYP2,3 families is differentially expressed in our lines, future experiment based on allele-specific hybridization probes or gene specific RT-PCR assays should be conducted.

### Lean mice display an improved profile of adiposity, plasma lipoproteins, plasma bile acids and liver triglycerides parameters

Food intake analysis demonstrated statistically non-significant differences between Lean and Fat mice (data not shown) supporting previous findings that Lean and Fat mice, despite the large phenotypic difference, consume equal amounts of food [24]. Lean line males compared to the Fat males exhibited significantly lower body fatness at 14-weeks of age across all dissected fat depots (Table 4). The relative weight (normalized to body weight) of abdominal, femoral, epididymal and mesenterial fat of Lean mice was approximately 5.4, 4.0, 7.0 and 3.5 fold lower compared to Fat line males, respectively. On the contrary, the relative heart weight was 1.34 fold higher in Lean versus Fat mice.

**Table 4:** Weights of fat depots and heart normalized to body weight in Lean and Fat mice

Parameter	Lean mice*	Fat mice*	No. of individuals
BODY WEIGHT at 14 weeks (g)	28.17 ± 1.15	41.76 ± 3.22*	L = 23; F = 24
ABDOMINAL FAT			
ratio (mg/g body wt)	1.86 ± 069	9.97 ± 1.08^a^	L = 43; F = 42
FEMORAL FAT			
ratio (mg/g body wt)	4.43 ± 1.2	17.67 ± 2.32 ^a^	L = 43; F = 42
EPIDIDYMAL FAT			
ratio (mg/g body wt)	3.34 ± 0.85	23.24 ± 3.12 ^a^	L = 43; F = 42
MESENTERIAL FAT			
ratio (mg/g body wt)	6.43 ± 1.26	22.33 ± 3.87 ^a^	L = 18; F = 15
HEART			
ratio (mg/g body wt)	6.13 ± 0.87	4.58 ± 0.69 ^a^	L = 43; F = 42

*Results are expressed as means ± S.D. Except for mesenterial fat and heart, means were calculated based on weighed left-sided fat depot (L; Lean mice, F; Fat mice, ^a ^*P *< 0.001).

**Figure 2:**
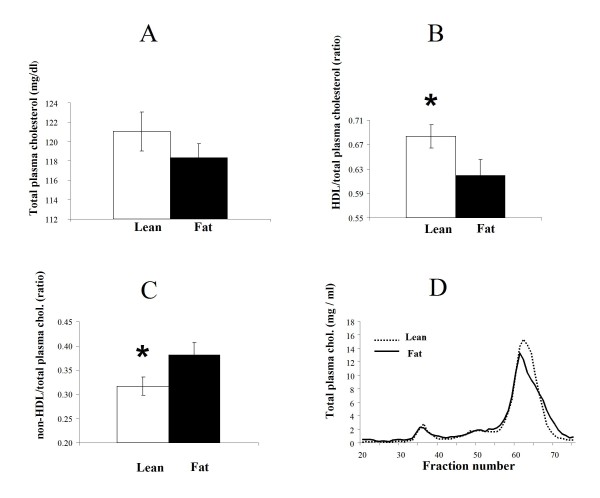
**Detailed plasma cholesterol analyses in the Lean and Fat mice**. **A**. Total plasma cholesterol concentrations. Although cholesterol biosynthesis genes showed increased expression in the liver of Lean mice (Table 3), total blood plasma cholesterol concentration was not significantly different to the Fat mice. Average concentrations of total plasma cholesterol (mg/dl) standard errors are displayed. **B**. Ratio of HDL-cholesterol to total plasma cholesterol. Beneficial HDL-cholesterol is significantly (*P < 0.05) increased in Lean mice. Average ratios and standard errors are displayed. **C**. Ratio of nonHDL-cholesterol to total plasma cholesterol. nonHDL-cholesterol was a derived parameter obtained by subtracting total plasma cholesterol from HDL-cholesterol. As HDL-cholesterol is markedly elevated in Lean mice (Figure 2B), consequently the Lean mice have significantly (*P < 0.05) lower ratio of atherogenic nonHDL-cholesterol to total plasma cholesterol. Average ratios and standard errors are displayed. **D**. FPLC analysis of total plasma lipoproteins. Cholesterol distribution among lipoproteins was obtained by separation of the major lipoprotein classes (VLDL, IDL+LDL, and HDL) by fast protein liquid chromatography (FPLC). This analysis determined in the Lean line a higher peak in the HDL fraction as well as a shift of HDL particles towards increased fraction elution number, suggesting that both the size and concentration of plasma HDL lipoproteins are elevated in Lean mice

Plasma was individually analyzed in Lean and Fat males for total plasma cholesterol (Figure 2A) and HDL/total plasma cholesterol ratio (Figure 2B). Non-HDL-cholesterol was calculated by subtracting total plasma cholesterol from HDL-cholesterol and the ratio of non-HDL-cholesterol/total plasma cholesterol was subsequently calculated (Figure 2C). The beneficial HDL-cholesterol was markedly elevated in Lean mice (Figure 2B) and consequently the Lean mice showed significantly lower ratios of atherogenic non-HDL-cholesterol versus total plasma cholesterol (Figure 2C). However, despite the higher expression level of genes involved in cholesterol biosynthesis in the Lean mice (Table 3), total plasma cholesterol concentration was not significantly different between the lines (Figure 2A). A follow up FPLC plasma analysis determined that the Lean line had a higher peak in the HDL fraction as well as a shift of the HDL curve suggesting that both the size and concentration of plasma HDL lipoproteins were elevated in Lean mice (Figure 2D).

**Figure 3:**
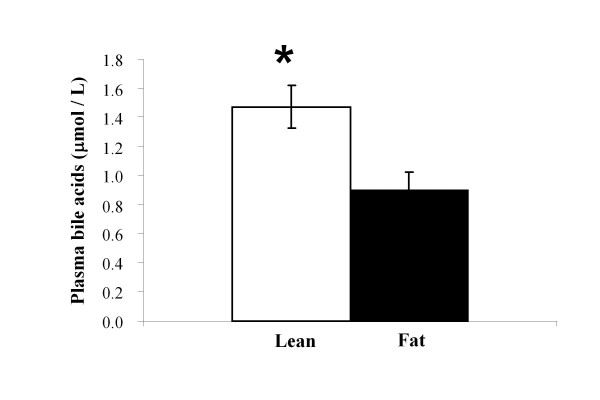
**Plasma bile acid concentration in the Lean and Fat mice**. Significantly higher concentrations (*P < 0.001) of total plasma bile acids were determined in the Lean mice. Average concentrations of plasma bile acids (μmol/L) with standard errors are displayed.

**Figure 4:**
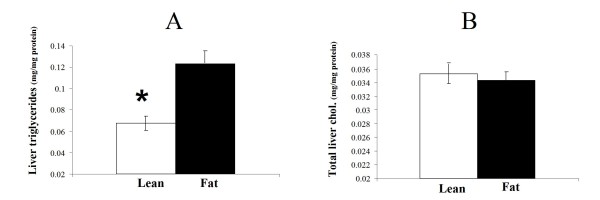
**Triglyceride and total cholesterol content in the livers of Lean and Fat mice**. **A**. Hepatic trygliceride levels. The liver of Lean mice contains significantly lower (*P < 0.05) amounts of triglycerides. Average values for mg of triglycerides per mg of liver proteins with standard errors are displayed. **B**. Total hepatic cholesterol levels. Despite that cholesterol biosynthesis genes showed increased mRNA expression in the liver of Lean mice (Table 3), total liver cholesterol does not differ significantly from the Fat line. Average values for mg of total cholesterol per mg of liver proteins with standard errors are displayed.

Significantly higher concentrations of total plasma bile acids were found in Lean mice (Figure 3) as well as lower hepatic triglyceride levels (Figure 4A). However, total liver cholesterol analysis resulted in statistically non-significant differences (Figure 4B) suggesting that this unlikely presents a driving effect.

### Lean mice show increased skeletal muscle oxidative capacity

**Figure 5:**
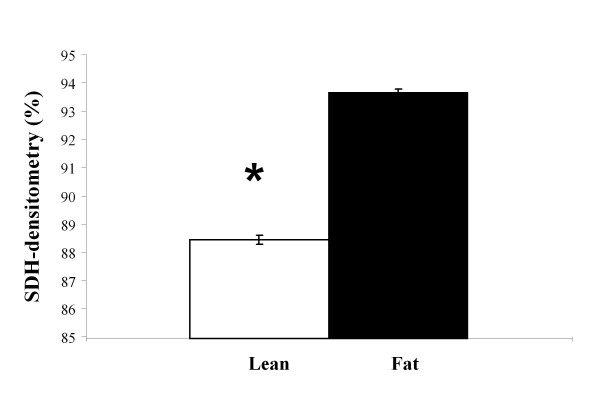
**SDH-activity in *m. gastrocnemius *muscle tissue of the Lean and Fat mice**. Striated muscle from *regio cruris *(*m. gastrocnemius*) was used to determine mitochondrial succinate dehydrogenase (SDH) activity levels. 12 of each Lean and Fat line mice were analysed and 150 muscle fibers per mouse were measured for densitometry. This histochemical assay demonstrated marked (*P < 0.001) increased level of SDH activity in muscle fibers of Lean mice suggesting pronounced increase in oxidative metabolism in this line. % densitometry with standard errors are displayed - lower transparency of muscle histological sections in the Lean mice resulted from higher SDH-enzyme activity which in turn gave lower % densitometry readings in the Lean line.

Analysis of *m. gastrocnemius *SDH-activity determined by histochemical reaction identified marked differences in oxidative capacity of skeletal muscle fibres from Fat versus Lean mice. Significantly higher levels of SDH activity were detected in muscle fibres of Lean mice (Figure 5, P < 0.001). Skeletal muscle interleukin 6 (*Il6*) mRNA level, a potent indicator of muscle physical activity, was examined using qRT-PCR. Lean mice muscle tissue displayed markedly elevated *Il6 *gene expression level (Table 2). Furthermore, a significant down- regulation of iodothyronine, type II deiodinase (*Dio2*) in *m. gastrocnemius *and *m. quadriceps *of Lean mice (Table 2) was determined.

## Discussion

The main objective of the present study was to identify candidate genes and molecular mechanisms responsible for obesity-resistance in the Lean line. Additionally, we aimed to investigate metabolic parameters of skeletal muscle and liver tissue and how they relate to blood lipoproteins, triglycerides, bile acids and liver transcriptome. Phenotypic characterization and search of differentially expressed genes was also carried out to identify novel positional candidate genes that map to the regions of quantitative trait loci determined in previous crosses between the Fat and Lean mice.

### Non-atherogenic lipoprotein profile in Lean mice

Plasma and liver cholesterol concentrations are, under balanced diet conditions, determined by the affluence from hepatic *de-novo *cholesterol biosynthesis. Additionally, excretion rate of cholesterol and bile acids also contribute to inter-individual variation of total plasma and liver cholesterol.

In the current experiment a large number of liver cholesterol biosynthesis genes were upregulated in the Lean line, but this was not reflected in an elevation of total liver and plasma cholesterol. This inconsistency could be explained in two ways: 1) either upregulation of cholesterol biosynthesis genes was not sufficient to increase the metabolic flow in cholesterol biosynthesis pathway that would result in an increased level of *de novo *hepatic cholesterol; 2) or that novel and excess hepatic cholesterol was excreted from the liver or metabolized to bile acids. Since we have not measured the metabolic rate of hepatic cholesterol biosynthesis or determined protein levels, we can not confirm yet that the increase in mRNA expression resulted in increased metabolic rate of cholesterol biosynthesis. Regulation of the cholesterol synthesis genes is under control of the SREBP signalling pathway [25] and upregulation observed in the Lean line is probably a result of activation of the SREBP transcription factor cascade. This is supported by our finding that *Insig-1 *and *Ldlr*, both being SREBP target genes, were up-regulated in the Lean mice. The hypothesis that cholesterol in the Lean mice would be excreted from the liver or metabolized to bile acids at an enhanced rate has limited supportive results. Higher efflux of cholesterol from the liver to bile is not likely since one of the genes involved in this process, *Abcg5*, is downregulated in the Lean mice. Increase in bile acid synthesis is plausible, since *Cyp8b1 *was up-regulated. However; *Cyp7a1*, a rate-limiting enzyme of bile acid synthesis, was not changed (data from RT-PCR not shown). Nevertheless, *Cyp7a1 *gene exhibits large interindividual differences in the level of expression and this hinders statistical significance. Also, we found that the nuclear receptor subfamily 5, group A, member 2 (*Nr5a2*, also known as LRH-1), a regulator of bile acid synthesis genes, is upregulated in Lean mice. In order to fully explain why up-regulation of cholesterol pathway genes in the Lean line did not lead to increased levels of total liver and plasma cholesterol, future detailed studies should be focused on protein and metabolic level of cholesterol management in Lean and Fat lines.

Although we did not find statistically significant differences in total liver and plasma cholesterol between the lines, the Lean mice livers did contain significantly less triglycerides per mg of liver tissue than the Fat mice - Lean mice had only about 50% of Fat mice triglyceride content (Figure 4). *Abcb11*, a gene overexpressed in the Lean line liver, is known to enhance the rate of triglyceride excretion from the liver [26,27]. Also, in a transgenic *Abcb11 *overexpression model [28], increased liver *Abcb11 *expression showed ameliorating effects on hepatic steatosis by increasing excretion of biliary phospholipids and cholesterol. It is possible therefore that upregulation of *Abcb11 *in the Lean mice might account for their diminished content of liver triglycerides. *Abcb11 *is also a strong positional candidate because it maps within a QTL *Fob1 *on chromosome 2, detected in our previous cross between Lean and Fat mice [17]. Also supportive is a study in humans detecting 30 mutations in the *ABCB11 *gene that were associated with different clinical phenotypes and triglyceride measurements [29]. Therefore, *Abcb11 *could be one of the potential causal allelic variants between the Lean and Fat mice, which can aid in explaining a part of the phenotype divergence between the strains. Elevated HDL and upregulation of some bile acids synthesis and transport genes suggests enhanced reverse cholesterol transport in the Lean line. As the cholesterol excretion rate is higher, Lean line compensates this by upregulating of endogenous cholesterol biosynthesis as demonstrated by the array experiment.

We established that Lean mice have significantly elevated plasma HDL. Interestingly, results from a previous study on humans imply that more exercise leads to increased total HDL and the average size of HDL particles [30]. FPLC analysis of plasma revealed similar observations - Lean mice have higher HDL plasma concentrations and also tend to have HDL particles of increased size. Interpretation for improved HDL profile in Lean mice can be further substantiated by genetic studies on HDL metabolism, where several QTLs were demonstrated to determine plasma HDL concentration, some also mapping to QTL regions detected in Fat and Lean mice [17,31]. It is therefore possible that some of the obesity QTLs identified in our previous genetic studies have pleiotropic effects affecting both body fat % and HDL levels in our polygenic animal model [17-19]. The explanation for the observed higher concentration of HDL and lower concentration of LDL-cholesterol in Lean mice may therefore lie in allelic differences in some of the HDL QTLs between the Fat and Lean mice.

### Plasma bile acids and changes in skeletal muscle metabolism

The bile acid pool in plasma is maintained by the enterohepatic recirculation. The uptake of bile acids that return to the liver after intestinal absorption is mainly mediated by a family of SLCO transporters (*Slco1a1, Slco1a4 , Slco10a1*) [27] whose expression can be modulated by plasma bile acids and cytokines. In our Lean mice down-regulation of genes encoding the aforementioned hepatic bile acid transporters along with significantly elevated concentrations of plasma bile acids was detected. Other studies have also demonstrated that bile acids can down-regulate expression of *Slco *genes and up-regulate *Abcb11 *[32], which is in agreement with our results obtained in the Lean line. Additional support comes from a study identifying significant dose-dependent mRNA suppression of *Slco1a1 *(*Oatp1*) and *Slco1a4 *(*Oatp2*) in mice treated with cytokine IL6 [33]. Detailed *in-vitro *and *in-vivo *studies defined IL6 as by far the most pronounced transcriptional regulator of, for example, *Slco1a1, Slco1a4 *and *Abcb11 *[33,34]. IL6, also termed as »exercise factor«, is highly expressed in contracting (exercising) skeletal muscle and is the most highly secreted systemic muscle cytokine during exercise [35]. The expression level of *Il6 *mRNA serves as a predictor of plasma IL6 protein [36]. In line with these reports, our analysis demonstrates high levels of expression of skeletal muscle *Il6 *mRNA in Lean mice. This increased *Il6 *expression in the Lean line could be a consequence of increased physical activity of Lean mice reported in our previous experiments [37]. Thus, relatively high IL6 plasma levels in Lean mice might suppress the expression of the *Slco *family of genes subsequently leading to the increase in plasma bile acids. However, future studies are needed to confirm that the muscle-derived cytokine IL6 in the Lean mice is directly responsible for down-regulation of *Slco *genes and increase in plasma bile acids.

Because Lean mice contain elevated levels of plasma bile acids we sought to interpret other potential metabolic actions involving bile acids. Elevated levels of plasma bile acids were found to lead to endocrine actions with consequent impact on whole body energy homeostasis [38]. This effect is elicited through the increased mRNA expression of type II iodothyronine deiodinase *(Dio2) *which was exemplified in the mouse brown adipose tissue and in human skeletal muscle myoblasts [38]. *Dio2 *expression was elevated in our Lean mice and also maps close to the *Fob2 *QTL, previously identified as one of four loci significantly contributing to the divergent body fat % in our polygenic mouse model [17]. In humans, polymorphisms at the *Dio2 *locus determines susceptibility to hypertension and diabetes [39,40]. Given the expression difference in the present study and its location to previously mapped QTL in the same mice, it is plausible that the *Dio2 *allelic variant also exists in this animal model, and may consequently determine differential expression and downstream consequences on phenotype in the Lean line.

The overall results of our current study are in line with our previous energy budget results which suggested that Lean mice expend more energy on physical activity [11] and our follow-up study demonstrating higher running wheel activity as well as higher exercise-independent posture allocation to more energy demanding positions (Non-Exercise Activity Thermogenesis; NEAT--standing, fidgeting, etc.) in Lean mice [37]. Increased oxidative capacity of muscle determined in the current study and resulting increased levels of *Il6 *expression may therefore indeed stem from higher physical and NEAT activities leading to downstream favourable effects on the liver transcriptome, cholesterol, bile acid, glucose and lipoprotein homeostasis.

However, the identified differences in liver transcriptome and other parameters and their associations with muscle metabolism and physical activity can explain only a part of a large phenotypic divergence between the Lean and Fat line. In a polygenic model it is expected that many alleles at different loci will contribute cumulatively to obesity resistance in various components of energy intake and energy expenditure. Since we showed in this study that Lean mice have similar food intake to the Fat line, we can exclude hyperphagia or genetic differences in energy intake as a major component for the phenotypic divergence between the lines. However, we have already shown earlier that thermoregulation [11] may also be increased in the Lean line and potential differences in resting metabolic rate can not be excluded. Therefore, more comprehensive experiments monitoring behavioural traits in combination with metabolic changes to measure differences in fuel oxidation will be needed to provide a better basis for future quantitative evaluations of energy expenditure components and fuel utilisation. Our current study reveals several candidate genes as well as important target tissues on which to base new targeted hypotheses to be tested in the future.

## Conclusions

Polygenic mouse models of leanness/obesity used in our study comprise complex obesity-resistant (Lean line) and obesity-susceptible (Fat line) gene networks. This study was designed to help identify candidate genes and reveal some metabolic mechanisms responsible for the phenotypic difference between these lines. Results of liver transcriptome analysis along with markers of oxidative capacity (SDH enzyme activity, *Il6 *expression) in skeletal muscle suggests of multiple tissue metabolic interactions in the obesity resistant Lean line. Candidate genes, particularly those mapping to previously detected QTL regions (*Abcb11 *and *Dio2*) between Lean and Fat mice, and new phenotypic parameters obtained in this study will give us leads to identify novel genes underlying this complex network. The results of the present study combined with previous findings help to elucidate, at least in part, a molecular basis for the contrasting phenotype between the Lean and Fat mice and substantiate a hypothesis concerning metabolic interactions between the physical activity, skeletal muscle and liver tissue. We demonstrate that the Lean mouse line is a potentially good model for identifying novel genes and mechanisms in obesity resistance.

## Authors' contributions

SH initiated, designed and co-ordinated the study, MS also participated in the design, performed animal experiments, RNA extraction, participated in the microarray hybridizations, qRT-PCR analyses, statistical analyses and drafted the manuscript; DR participated in the co-ordination of microarray studies and its experimental design, TR participated in the microarray and qRT-PCR experiments, PJ performed analysis of microarray data, CF and BS performed lipoprotein analyses and helped interpret phenotypic results, GF was involved in the experiment examining oxidative capacity of the muscle fibres. All authors contributed scientifically to the content of this study and have read and approved the final version of submitted manuscript.

## Supplementary Material

Additional file 1 **List of genes contained on the custom Steroltalk v2 microarray prepared for this study**. Click here for file
