# Supplementary material for: Obesity resistant mechanisms in the Lean polygenic mouse model as indicated by liver transcriptome and expression of selected genes in skeletal muscle
Source: BMC Genomics. 2011 Feb 3;12:96. doi: 10.1186/1471-2164-12-96 (PMC3044672; doi:10.1186/1471-2164-12-96)
Supplement: Additional file 1 — List of genes contained on the custom Steroltalk v2 microarray prepared for this study. [file 1471-2164-12-96-S1.PDF]

Supplemental Table 1: List of genes contained on the custom Steroltalk v2 microarray prepared for this study

Gene	GenBank No.	Description	Functional groups
Ch25h	NM_009890	Cholesterol 25-hydroxylase	Bile acid synthesis
Cyp27a1	NM_024264	Cytochrome P450, family 27, subfamily a, polypeptide 1	Bile acid synthesis
Cyp27b1	AB006034	Cytochrome P450, family 27, subfamily b, polypeptide 1	Bile acid synthesis
Cyp39a1	AF237981	Cytochrome P450, family 39, subfamily a, polypeptide 1	Bile acid synthesis
Cyp46a1	NM_010010	Cytochrome P450, family 46, subfamily a, polypeptide 1	Bile acid synthesis
Cyp7a1	NM_007824	Cytochrome P450, family 7, subfamily a, polypeptide 1	Bile acid synthesis
Cyp7b1	BC038810	Cytochrome P450, family 7, subfamily b, polypeptide 1	Bile acid synthesis
Cyp8b1	NM_010012	Cytochrome P450, family 8, subfamily b, polypeptide 1	Bile acid synthesis
G6Pase	NM_008061	Glucose-6-phosphatase catalytic	Carbohydrate metabolism
Gck	BC011139	Glucokinase	Carbohydrate metabolism
Gsk3b	NM_019827	Glycogen synthase kinase 3 beta	Carbohydrate metabolism
Hk1	BC072628	Hexokinase 1	Carbohydrate metabolism
Hk2	BC054472	Hexokinase 2	Carbohydrate metabolism
Pck1	BC037629	Phosphoenolpyruvate carboxykinase 1, cytosolic	Carbohydrate metabolism
Pdhb	NM_024221	Pyruvate dehydrogenase (lipoamide) beta	Carbohydrate metabolism
Pfkm	NM_021514	Phosphofructokinase, muscle	Carbohydrate metabolism
Pklr	NM_013631	Pyruvate kinase liver and red blood cell	Carbohydrate metabolism
Pkm2	BC016619	Pyruvate kinase, muscle	Carbohydrate metabolism
Adipoq	BC028770	Adiponectin, C1Q and collagen domain containing	Cell signaling
Adipor1	NM_028320	Adiponectin receptor 1	Cell signaling
Adipor2	NM_197985	Adiponectin receptor 2	Cell signaling
Arntl2	AY005163	Aryl hydrocarbon receptor nuclear translocator-like 2	Cell signaling
Camk1d	NM_177343	Calcium/calmodulin-dependent protein kinase ID	Cell signaling
Camkk1	AF117384	Calcium/calmodulin-dependent protein kinase kinase 1, alpha	Cell signaling
Casp3	NM_009810	Caspase 3, apoptosis-related cysteine peptidase	Cell signaling
Casp4	NM_007609	Caspase 4, apoptosis-related cysteine peptidase	Cell signaling
Cav1	NM_007616	Caveolin, caveolae protein 1	Cell signaling
Egr1	M20157	Early growth response 1	Cell signaling
Gckr	NM_144909	Glucokinase regulatory protein	Cell signaling
Ghrl	NM_021488	Ghrelin	Cell signaling
Igf1	NM_010512	Insulin-like growth factor 1	Cell signaling
Irs1	AK142242	Insulin receptor substrate 1	Cell signaling
Irs2	XM_357863	Insulin receptor substrate 2	Cell signaling
Jak1	BC031297	Janus kinase 1	Cell signaling
Jak2	BC054807	Janus kinase 2	Cell signaling
Jun	BC002081	Jun oncogene	Cell signaling
Lep	U18812	Leptin	Cell signaling
Lepr	BC082551	Leptin receptor	Cell signaling
Lipe	NM_010719	Lipase, hormone sensitive	Cell signaling
Mapk8	BC053027	Mitogen activated protein kinase 8	Cell signaling
Mapk9	BC028341	Mitogen activated protein kinase 9	Cell signaling
Msr1	BC003814	Macrophage scavenger receptor 1	Cell signaling
Msr1	AF203781	Macrophage scavenger receptor 1	Cell signaling
Nfkbia	BC046754	Nuclear factor of kappa light chain gene enhancer in B-cells inhibitor, alpha	Cell signaling
Nfkbie	BC030923	Nuclear factor of kappa light polypeptide gene enhancer in B-cells inhibitor, epsilon	Cell signaling
Ogt	BC057319	O-linked N-acetylglucosamine (GlcNAc) transferase	Cell signaling
Oprs1	NM_011014	Opioid receptor, sigma 1	Cell signaling

Gene	GenBank No.	Description	Functional groups
Pik3cg	NM_020272	Phosphoinositide-3-kinase, catalytic, gamma polypeptide	Cell signaling
Ppp1r3c	NM_016854	Protein phosphatase 1, regulatory (inhibitor) subunit 3C	Cell signaling
Prkaa1	NM_001013367	Protein kinase, AMP-activated, alpha 1 catalytic subunit	Cell signaling
Prkaca	BC003238	Protein kinase, cAMP dependent, catalytic, alpha	Cell signaling
Prkca	BC096493	Protein kinase C, alpha	Cell signaling
Prkcb1	NM_008855	Protein kinase C, beta 1	Cell signaling
Retn	NM_022984	Resistin	Cell signaling
Serping1	BC002026	Serine (or cysteine) peptidase inhibitor, clade G, member 1	Cell signaling
Stat3	BC003806	Signal transducer and activator of transcription 3	Cell signaling
Sumo1	BC082566	SMT3 suppressor of mif two 3 homolog 1 (yeast)	Cell signaling
Sumo2	BC017522	SMT3 suppressor of mif two 3 homolog 2 (yeast)	Cell signaling
Cyp51	BC031813	Cytochrome P450, family 51	Cholesterol biosynthesis
Dhcr24	BC019797	24-dehydrocholesterol reductase	Cholesterol biosynthesis
Dhcr7	BC006854	7-dehydrocholesterol reductase	Cholesterol biosynthesis
Ebp	NM_007898	Emopamil binding protein	Cholesterol biosynthesis
Fdft1	NM_010191	Farnesyl diphosphate farnesyl transferase 1, Squalene synthase	Cholesterol biosynthesis
Fdps	NM_134469	Farnesyl diphosphate synthase 1	Cholesterol biosynthesis
Hmgcr	NM_008255	3-hydroxy-3-methylglutaryl-Coenzyme A reductase	Cholesterol biosynthesis
Hmgcs1	NM_145942	3-hydroxy-3-methylglutaryl-Coenzyme A synthase 1	Cholesterol biosynthesis
Idi1	NM_177960	Isopentenyl-diphosphate delta isomerase	Cholesterol biosynthesis
Lss	NM_146006	Lanosterol synthase	Cholesterol biosynthesis
Mvd	NM_138656	Mevalonate (diphospho) decarboxylase	Cholesterol biosynthesis
Mvk	NM_023556	Mevalonate kinase	Cholesterol biosynthesis
Nsdhl	BC019945	NAD(P) dependent steroid dehydrogenase-like	Cholesterol biosynthesis
Pmvk	NM_026784	Phosphomevalonate kinase	Cholesterol biosynthesis
Sc4mol	NM_025436	Sterol-C4-methyl oxidase-like	Cholesterol biosynthesis
Sc5d	BC024132	Sterol-C5-desaturase homolog	Cholesterol biosynthesis
Sqle	BC042781	Squalene epoxidase	Cholesterol biosynthesis
Arntl	BC011080	Aryl hydrocarbon receptor nuclear translocator-like	Circadian regulation
Clock	AF000998	Circadian locomoter output cycles kaput	Circadian regulation
Cry1	NM_007771	Cryptochrome 1 (photolyase-like)	Circadian regulation
Cry2	NM_009963	Cryptochrome 2 (photolyase-like)	Circadian regulation
Dbp	BC018323	D site albumin promoter binding protein	Circadian regulation
Per1	NM_011065	Period homolog 1 (Drosophila)	Circadian regulation
Per2	NM_011066	Period homolog 2 (Drosophila)	Circadian regulation
Per3	NM_011067	Period homolog 3 (Drosophila)	Circadian regulation
Timeless	BC058641	Timeless homolog (Drosophila)	Circadian regulation
Cyp1a1	NM_009992	Cytochrome P450, family 1, subfamily a, polypeptide 1	Drug metabolism
Cyp1a2	NM_009993	Cytochrome P450, family 1, subfamily a, polypeptide 2	Drug metabolism
Cyp1b1	BC050063	Cytochrome P450, family 1, subfamily b, polypeptide 1	Drug metabolism
Cyp2a12	NM_133657	Cytochrome P450, family 2, subfamily a, polypeptide 12	Drug metabolism
Cyp2a4	BC011233	Cytochrome P450, family 2, subfamily a, polypeptide 4	Drug metabolism
Cyp2b10	AK028103	Cytochrome P450, family 2, subfamily b, polypeptide 10	Drug metabolism
Cyp2b13	NM_007813	Cytochrome P450, family 2, subfamily b, polypeptide 13	Drug metabolism
Cyp2b9	NM_010000	Cytochrome P450, family 2, subfamily b, polypeptide 9	Drug metabolism
Cyp2c40	NM_010004	Cytochrome P450, family 2, subfamily c, polypeptide 40	Drug metabolism
Cyp2d22	NM_019823	Cytochrome P450, family 2, subfamily d, polypeptide 22	Drug metabolism
Cyp2e1	NM_021282	Cytochrome P450, family 2, subfamily e, polypeptide 1	Drug metabolism
Cyp2f2	NM_007817	Cytochrome P450, family 2, subfamily f, polypeptide 2	Drug metabolism

Gene	GenBank No.	Description	Functional groups
Cyp2g1	NM_013809	Cytochrome P450, family 2, subfamily g, polypeptide 1	Drug metabolism
Cyp2j5	NM_010007	Cytochrome P450, family 2, subfamily j, polypeptide 5	Drug metabolism
Cyp2j6	U62295	Cytochrome P450, family 2, subfamily j, polypeptide 6	Drug metabolism
Cyp3a11	NM_007818	Cytochrome P450, family 3, subfamily a, polypeptide 11	Drug metabolism
Cyp3a13	NM_007819	Cytochrome P450, family 3, subfamily a, polypeptide 13	Drug metabolism
Cyp3a25	BC028855	Cytochrome P450, family 3, subfamily a, polypeptide 25	Drug metabolism
Cyp3a41	NM_017396	Cytochrome P450, family 3, subfamily a, polypeptide 41	Drug metabolism
Acss2	NM_019811	Acyl-CoA synthetase short-chain family member 2	Fatty acid metabolism
Cpt1a	BC038395	Carnitine palmitoyltransferase 1a, liver	Fatty acid metabolism
Cyp4f14	NM_022434	Cytochrome P450, family 4, subfamily f, polypeptide 14	Fatty acid metabolism
Fasn	BC046513	Fatty acid synthase	Fatty acid metabolism
Hmgcl	BC025440	3-hydroxy-3-methylglutaryl-Coenzyme A lyase	Fatty acid metabolism
Lip1	NM_021460	Lysosomal acid lipase 1	Fatty acid metabolism
Pla2g6	BC003487	Phospholipase A2, group VI	Fatty acid metabolism
Ptgis	NM_008968	Prostaglandin I2 (prostacyclin) synthase	Fatty acid metabolism
Scd1	BC007474	Stearoyl-Coenzyme A desaturase 1	Fatty acid metabolism
Tbxas1	NM_011539	Thromboxane A synthase 1, platelet	Fatty acid metabolism
Alas1	NM_020559	Aminolevulinic acid synthase 1	Heme metabolism
Hmox1	BC010757	Heme oxygenase (decycling) 1	Heme metabolism
Hpxn	BC019901	Hemopexin	Heme metabolism
Actb	NM_007393	Actin, beta, cytoplasmic	Housekeeping genes
Actb	NM_007393	Actin, beta, cytoplasmic	Housekeeping genes
Gapdh	NM_008084	Glyceraldehyde-3-phosphate dehydrogenase	Housekeeping genes
Gapdh	NM_008084	Glyceraldehyde-3-phosphate dehydrogenase	Housekeeping genes
Gapdh	NM_008084	Glyceraldehyde-3-phosphate dehydrogenase	Housekeeping genes
Ppia	NM_008907	Peptidylprolyl isomerase A	Housekeeping genes
Rps6ka5	AK030051	Ribosomal protein S6 kinase, polypeptide 5	Housekeeping genes
Apoa1	NM_009692	Apolipopoprotein A-I	Lipid transport
Apoa2	BC031786	Apolipoprotein A-II	Lipid transport
Apoa4	BC010769	Apolipoprotein A-IV	Lipid transport
Apoa5	BC011198	Apolipoprotein A-V	Lipid transport
Apob	XM_137955	Apolipoprotein B	Lipid transport
Apoc1	BC094638	Apolipoprotein C-I	Lipid transport
Apoc2	NM_009695	Apolipoprotein C-II	Lipid transport
Apoc3	BC021776	Apolipoprotein C-III	Lipid transport
Apoe	BC028816	Apolipoprotein E	Lipid transport
Ldlr	BC019207	Low density lipoprotein receptor	Lipid transport
Ldlrap1	NM_145554	Low density lipoprotein receptor adaptor protein 1	Lipid transport
Ar	M37890	Androgen receptor	Nuclear receptor superfamily
Esr1	NM_007956	Estrogen receptor 1 (alpha)	Nuclear receptor superfamily
Esr2	NM_010157	Estrogen receptor 2 (beta)	Nuclear receptor superfamily
Esrra	NM_007953	Estrogen related receptor, alpha	Nuclear receptor superfamily
Esrrb	NM_011934	Estrogen related receptor, beta	Nuclear receptor superfamily
Esrrg	NM_011935	Estrogen-related receptor gamma	Nuclear receptor superfamily
Hnf4a	NM_008261	Hepatic nuclear factor 4, alpha	Nuclear receptor superfamily
Hnf4g	NM_013920	Hepatocyte nuclear factor 4, gamma	Nuclear receptor superfamily
Nr0b1	NM_007430	Nuclear receptor subfamily 0, group B, member 1	Nuclear receptor superfamily
Nr0b2	NM_011850	Nuclear receptor subfamily 0, group B, member 2	Nuclear receptor superfamily
Nr1d2	NM_011584	Nuclear receptor subfamily 1, group D, member 2	Nuclear receptor superfamily

Gene	GenBank No.	Description	Functional groups
Nr1h2	NM_009473	liver X receptor beta	Nuclear receptor superfamily
Nr1h4	NM_009108	Nuclear receptor subfamily 1, group H, member 4	Nuclear receptor superfamily
Nr1i2	NM_010936	Nuclear receptor subfamily 1, group I, member 2	Nuclear receptor superfamily
Nr1i3	NM_009803	Nuclear receptor subfamily 1, group I, member 3	Nuclear receptor superfamily
Nr2c1	NM_011629	Nuclear receptor subfamily 2, group C, member 1	Nuclear receptor superfamily
Nr2c2	NM_011630	Nuclear receptor subfamily 2, group C, member 2	Nuclear receptor superfamily
Nr2e1	NM_152229	Nuclear receptor subfamily 2, group E, member 1	Nuclear receptor superfamily
Nr2e3	NM_013708	Nuclear receptor subfamily 2, group E, member 3	Nuclear receptor superfamily
Nr2f1	NM_010151	Nuclear receptor subfamily 2, group F, member 1	Nuclear receptor superfamily
Nr2f2	NM_009697	Nuclear receptor subfamily 2, group F, member 2	Nuclear receptor superfamily
Nr2f6	NM_010150	Nuclear receptor subfamily 2, group F, member 6	Nuclear receptor superfamily
Nr3c1	NM_008173	Nuclear receptor subfamily 3, group C, member 1	Nuclear receptor superfamily
Nr4a1	NM_010444	Nuclear receptor subfamily 4, group A, member 1	Nuclear receptor superfamily
Nr4a2	NM_013613	Nuclear receptor subfamily 4, group A, member 2	Nuclear receptor superfamily
Nr4a3	NM_015743	Nuclear receptor subfamily 4, group A, member 3	Nuclear receptor superfamily
Nr5a1	NM_139051	Nuclear receptor subfamily 5, group A, member 1	Nuclear receptor superfamily
Nr5a2	NM_030676	Nuclear receptor subfamily 5, group A, member 2	Nuclear receptor superfamily
Nr6a1	AF390896	Nuclear receptor subfamily 6, group A, member 1	Nuclear receptor superfamily
Pgr	NM_008829	Progesterone receptor	Nuclear receptor superfamily
Ppara	NM_011144	Peroxisome proliferator activated receptor alpha	Nuclear receptor superfamily
Ppard	NM_011145	Peroxisome proliferator activator receptor delta	Nuclear receptor superfamily
Pparg	NM_011146	Peroxisome proliferator activated receptor gamma	Nuclear receptor superfamily
Rara	NM_009024	Retinoic acid receptor, alpha	Nuclear receptor superfamily
Rarb	NM_011243	Retinoic acid receptor, beta	Nuclear receptor superfamily
Rarg	NM_011244	Retinoic acid receptor, gamma	Nuclear receptor superfamily
Rora	NM_013646	RAR-related orphan receptor alpha	Nuclear receptor superfamily
Rorb	NM_146095	RAR-related orphan receptor beta	Nuclear receptor superfamily
Rorc	NM_011281	RAR-related orphan receptor gamma	Nuclear receptor superfamily
Rxra	NM_011305	Retinoid X receptor alpha	Nuclear receptor superfamily
Rxrb	NM_011306	retinoid X receptor beta	Nuclear receptor superfamily
Rxrg	NM_009107	Retinoid X receptor gamma	Nuclear receptor superfamily
Thra	NM_178060	thyroid hormone receptor alpha	Nuclear receptor superfamily
Thrb	NM_009380	thyroid hormone receptor beta	Nuclear receptor superfamily
Vdr	NM_009504	Vitamin D receptor	Nuclear receptor superfamily
Acat2	NM_009338	Acetyl-Coenzyme A acetyltransferase 2	Other
Cyp20a1	BC049147	Cytochrome P450, family 20, subfamily A, polypeptide 1	Other
Cyp24a1	NM_009996	Cytochrome P450, family 24, subfamily A, polypeptide 1	Other
Cyp26a1	NM_007811	Cytochrome P450, family 26, subfamily a, polypeptide 1	Other
Cyp26b1	NM_175475	Cytochrome P450, family 26, subfamily b, polypeptide 1	Other
Cyp4b1	NM_007823	Cytochrome P450, family 4, subfamily b, polypeptide 1	Other
Gfpt2	BC031928	Glutamine fructose-6-phosphate transaminase 2	Other
Icam1	BC008626	Intercellular adhesion molecule	Other
Nos1	BC066101	Nitric oxide synthase 1, neuronal	Other
Nos2	BC062378	Nitric oxide synthase 2, inducible, macrophage	Other
Nos3	BC052636	Nitric oxide synthase 3, endothelial cell	Other
Orm1	BC012725	Orosomucoid 1	Other
Pon1	BC012706	Paraoxonase 1	Other
Pon2	NM_183308	Paraoxonase 2	Other
Pten	BC021445	Phosphatase and tensin homolog	Other

Gene	GenBank No.	Description	Functional groups
Scara3	BC051636	Scavenger receptor class A, member 3	Other
Scarb1	NM_016741	Scavenger receptor class B, member 1	Other
Scarb2	BC029073	Scavenger receptor class B, member 2	Other
Scp2	BC018384	Sterol carrier protein 2, liver	Other
Soat1	NM_009230	Sterol O-acyltransferase 1	Other
Soat2	BC025931	Sterol O-acyltransferase 2	Other
Star	AK054470	Steroidogenic acute regulatory protein	Other
Uap1	BC016406	UDP-N-acetylglucosamine pyrophosphorylase 1	Other
Ucp2	NM_011671	Uncoupling protein 2 (mitochondrial, proton carrier)	Other
Vcam1	BC029823	Vascular cell adhesion molecule 1	Other
Alb1	BC049971	Albumin 1	Serum proteins
Apcs	BC061125	Serum amyloid P-component	Serum proteins
C2	BC011086	Complement component 2 (within H-2S)	Serum proteins
C3	BC043338	Complement component 3	Serum proteins
C4b	BC067409	Complement component 4B (Childo blood group)	Serum proteins
C4bp	NM_007576	Complement component 4 binding protein	Serum proteins
C9	BC011137	Complement component 9	Serum proteins
Crp	NM_007768	C-reactive protein, pentraxin-related	Serum proteins
Fgb	NM_181849	Fibrinogen, B beta polypeptide	Serum proteins
Hc	M35525	Hemolytic complement	Serum proteins
Saa1	BC087933	Serum amyloid A 1	Serum proteins
Saa2	BC024606	Serum amyloid A 2	Serum proteins
Saa3	BC055885	Serum amyloid A 3	Serum proteins
Saa4	BC019212	Serum amyloid A 4	Serum proteins
Insig1	NM_153526	Insulin induced gene 1	SREBF signaling pathway
Insig2	BC023874	Insulin induced gene 2	SREBF signaling pathway
Mbtps1	NM_019709	Membrane-bound transcription factor peptidase, site 1	SREBF signaling pathway
Mbtps2	NM_172307	Membrane-bound transcription factor peptidase, site 2	SREBF signaling pathway
Scap	NM_001001144	SREBP cleavage activating protein	SREBF signaling pathway
Srebf1	NM_011480	Sterol regulatory element binding factor 1	SREBF signaling pathway
Srebf2	NM_033218	Sterol regulatory element binding factor 2	SREBF signaling pathway
Cyp11a1	NM_019779	Cytochrome P450, family 11, subfamily a, polypeptide 1	Steroid synthesis
Cyp11b2	NM_009991	Cytochrome P450, family 11, subfamily b, polypeptide 2	Steroid synthesis
Cyp17a1	NM_007809	Cytochrome P450, family 17, subfamily a, polypeptide 1	Steroid synthesis
Cyp19a1	NM_007810	Cytochrome P450, family 19, subfamily a, polypeptide 1	Steroid synthesis
Cyp21a1	NM_009995	Cytochrome P450, family 21, subfamily a, polypeptide 1	Steroid synthesis
Cebpa	BC028890	CCAAT/enhancer binding protein (C/EBP), alpha	Transcription regulators
Cebpd	X61800	CCAAT/enhancer binding protein (C/EBP), delta	Transcription regulators
Cebpg	BC011319	CCAAT/enhancer binding protein (C/EBP), gamma	Transcription regulators
Cebpz	NM_009882	CCAAT/Enhancer Binding Protein Zeta	Transcription regulators
Creb1	BC021649	CAMP responsive element binding protein 1	Transcription regulators
Crebbp	NM_001025432	CREB binding protein	Transcription regulators
Crem	M60285	CAMP responsive element modulator	Transcription regulators
Crem	M60285	CAMP responsive element modulator, transcript tau	Transcription regulators
Fhl5	AF083394	Four and a half LIM domains 5, also testis CREM activator	Transcription regulators
Fos	NM_010234	FBJ osteosarcoma oncogene	Transcription regulators
Foxa1	X74936	Forkhead box A1	Transcription regulators
Foxa2	NM_010446	Forkhead box A2	Transcription regulators
Foxo1	NM_019739	Forkhead box O1	Transcription regulators

**Gene**	**GenBank No.**	**Description**	**Functional groups**
Hif1a	BC026139	Hypoxia inducible factor 1, alpha subunit	Transcription regulators
Mlxipl	NM_021455	MLX interacting protein-like, old Wbscr14 or Chrebp	Transcription regulators
Ncoa1	BC068177	Nuclear receptor coactivator 1	Transcription regulators
Ncor1	NM_011308	Nuclear receptor co-repressor 1	Transcription regulators
Ncor2	AF113001	Nuclear receptor co-repressor 2	Transcription regulators
Nrf1	BC005410	Nuclear respiratory factor 1	Transcription regulators
Pcaf	BC082581	P300/CBP-associated factor	Transcription regulators
Ppargc1a	NM_008904	Peroxisome proliferative activated receptor, gamma, coactivator 1 alpha	Transcription regulators
Ppargc1a	NM_008904	Peroxisome proliferative activated receptor, gamma, coactivator 1 alpha	Transcription regulators
Ppargc1b	NM_133249	Peroxisome proliferative activated receptor, gamma, coactivator 1 beta	Transcription regulators
Sirt1	NM_019812	Sirtuin 1, silent mating type information regulation 2, homolog 1	Transcription regulators
Sp1	AF062566	Trans-acting transcription factor 1, Specificity protein 1	Transcription regulators
Tbp	BC012685	TATA box binding protein	Transcription regulators
Tcf1	NM_009327	Transcription factor 1	Transcription regulators
Trp53	BC005448	Transformation related protein 53	Transcription regulators
Abca1	NM_013454	ATP-binding cassette, sub-family A (ABC1), member 1	Transporters
Abcb11	NM_021022	ATP-binding cassette, sub-family B (MDR/TAP), member 11	Transporters
Abcb1a	NM_011076	ATP-binding cassette, sub-family B (MDR/TAP), member 1A	Transporters
Abcb1b	NM_011075	ATP-binding cassette, sub-family B (MDR/TAP), member 1B	Transporters
Abcb4	NM_008830	ATP-binding cassette, sub-family B (MDR/TAP), member 4	Transporters
Abcb7	BC035534	ATP-binding cassette, sub-family B (MDR/TAP), member 7	Transporters
Abcc1	NM_008576	ATP-binding cassette, sub-family C (CFTR/MRP), member 1	Transporters
Abcc2	NM_013806	ATP-binding cassette, sub-family C (CFTR/MRP), member 2	Transporters
Abcc3	NM_029600	ATP-binding cassette, sub-family C (CFTR/MRP), member 3	Transporters
Abcg1	AF323659	ATP-binding cassette, sub-family G (WHITE), member 1	Transporters
Abcg4	AJ426047	ATP-binding cassette, sub-family G (WHITE), member 4	Transporters
Abcg5	NM_031884	ATP-binding cassette, sub-family G (WHITE), member 5	Transporters
Abcg8	NM_026180	ATP-binding cassette, sub-family G (WHITE), member 8	Transporters
Fabp6	NM_008375	Fatty acid binding protein 6, ileal (gastrotropin)	Transporters
Slc10a1	BC021154	Solute carrier family 10, member 1	Transporters
Slc10a1	BC094023	Solute carrier family 10 (sodium/bile acid cotransporter family), member 1	Transporters
Slc10a2	NM_011388	Solute carrier family 10, member 2	Transporters
Slc2a1	BC055340	Solute carrier family 2 (facilitated glucose transporter), member 1	Transporters
Slc2a4	BC014282	Solute carrier family 2 (facilitated glucose transporter), member 4	Transporters
Slc2a8	BC090993	Solute carrier family 2, (facilitated glucose transporter), member 8	Transporters
Slco1a1	AY195868	Solute carrier organic anion transporter family, member 1a1	Transporters
Slco1a4	NM_030687	Solute carrier organic anion transporter family, member 1a4	Transporters
Slco1a5	AF240694	Solute carrier organic anion transporter family, member 1a5	Transporters
Slco1b2	NM_020495	Solute carrier organic anion transporter family, member 1b2	Transporters
Slco1c1	AY007379	Solute carrier organic anion transporter family, member 1c1	Transporters
Slco2b1	BC096485	Solute carrier organic anion transporter family, member 2b1	Transporters
